# Pseudoaneurysm of the anterior tibial artery after interlocking tibial nailing: an unexpected complication

**DOI:** 10.1186/s40001-016-0231-z

**Published:** 2016-09-29

**Authors:** F. Greve, M. Crönlein, M. Beirer, C. Kirchhoff, P. Biberthaler, K. F. Braun

**Affiliations:** Klinik und Poliklinik für Unfallchirurgie, Klinikum Rechts der Isar, Technische Universität München, Ismaninger Str. 22, 81675 Munich, Germany

**Keywords:** Tibial nailing, Pseudoaneurysm, False aneurysm, Complications, Peroneal lesion

## Abstract

Anterior tibial pseudoaneurysm is a rare complication after interlocking screw insertion in tibial nailing. We present the case of a 28-year-old male patient with this complication with a 6-week delay after tibial nailing of a right tibial fracture type 42-A1 of the Association for the Study of Internal Fixation (AO/ASIF) classification. On presentation to our emergency department, the patient’s complaints were solemnly intermittent pain and occasional swelling of his proximal lower leg. Deep vein thrombosis, compartment syndrome, and implant dislocation were ruled out, and the patient was discharged after his symptoms improved without further intervention. Four weeks later, the patient was readmitted for similar symptoms. A computed tomography (CT) angiography then revealed a pseudoaneurysm of the anterior tibial artery at the level of the proximal interlocking screw insertion. Aneurysmal sac excision with vessel repair was performed while reconstructing the additional dislocated proximal fibular fracture using standard AO/ASIF plating. Postoperatively, sufficient flow through the repaired vessel was documented using Doppler ultrasound and CT angiography. However, the patient sustained a temporal damage to the peroneal nerve after surgery. This case highlights the risk of a pseudoaneurysm of the anterior tibial artery after interlocking screw insertion as a rare but major complication of a routine surgical procedure. Early ultrasound diagnostics, CT angiography, or magnetic resonance (MR) angiogram should be performed to prevent the delay in diagnosis and treatment of such complications.

## Background

The therapeutic gold standard for diaphyseal tibial fractures is closed reduction with intramedullary nailing. This procedure is considered a routine and relatively safe intervention. Postoperative complications are rare and include infections, delayed fracture healing [1], and compartment syndrome [2, 3]. Formation of pseudoaneurysms are rarely observed and can occur either as a direct result of the initial trauma to the extremity [4–6] or being caused iatrogenically [7–13]. The following case outlines the clinical presentation of a pseudoaneurysm of the anterior tibial artery and highlights the importance of early diagnosis and therapeutic intervention in this rare and thus easily overlooked postoperative complication in tibial fractures.

## Case presentation

**Fig. 1:**
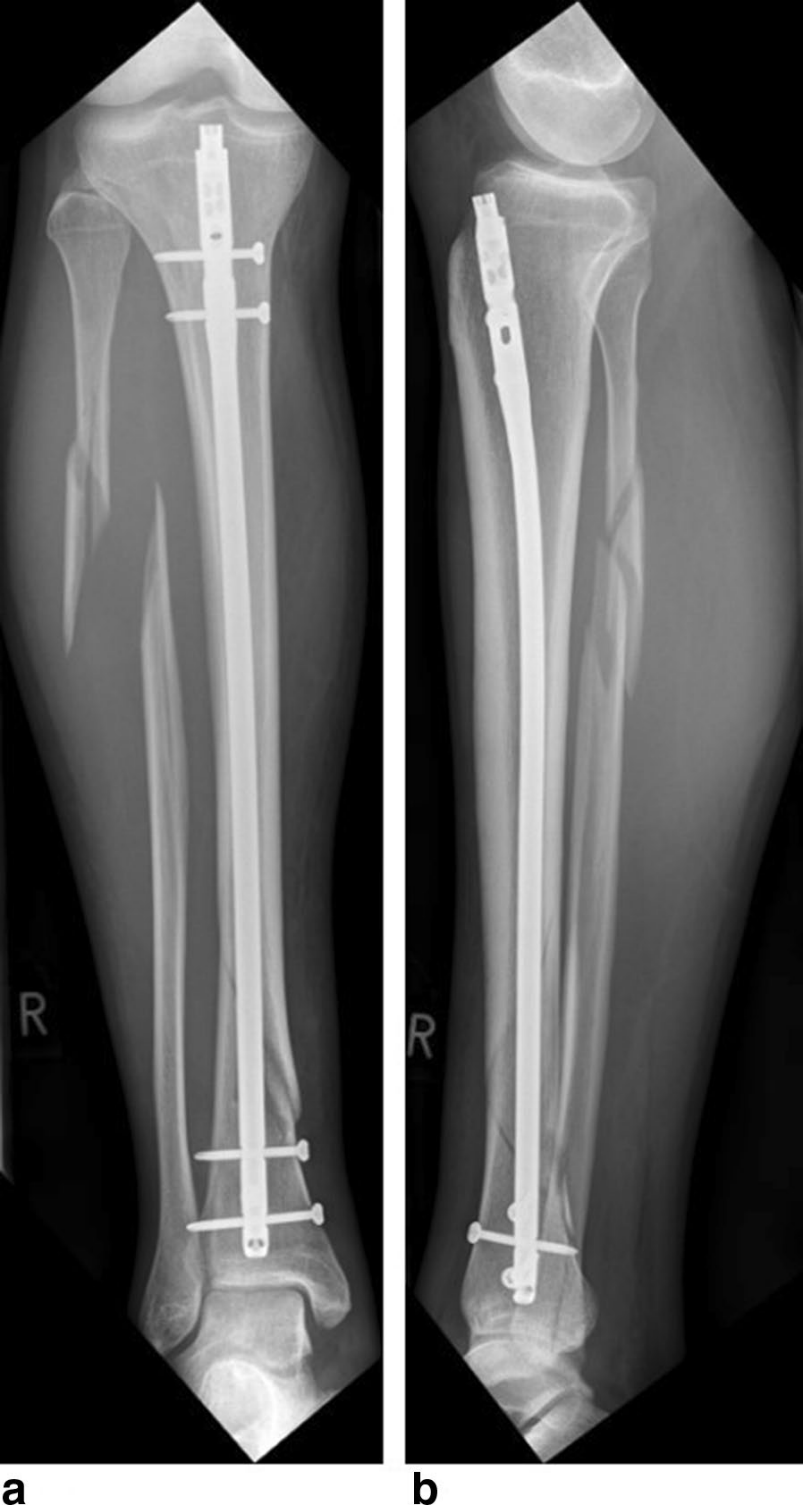
Anterio-posterior (**a**) and medio-lateral (**b**) radiographs of the right lower leg showing fracture reduction

A 28-year-old male patient was diagnosed with a complete right lower leg fracture (AO/ASIF 42-B1) after a skiing accident. The fracture was reduced using an Expert Tibia Nail (Synthes, Switzerland) in Austria without reduction of the fibula fracture. Two proximal medial–lateral, two distal medial–lateral, and one distal anterior–posterior interlocking screws (Fig. 1) were inserted. After an uncomplicated initial postoperative course and normal mobility, the patient presented with intermittent edema of the proximal lateral lower leg 6 weeks after surgery. Deep vein thrombosis was ruled out using ultrasound, and the patient was admitted for surveillance of a potential compartment syndrome. In addition, a distal interlocking screw was removed due to its close proximity to the distal tibial–fibular joint. The patient was discharged 3 days later after his symptoms had improved.

**Fig. 2:**
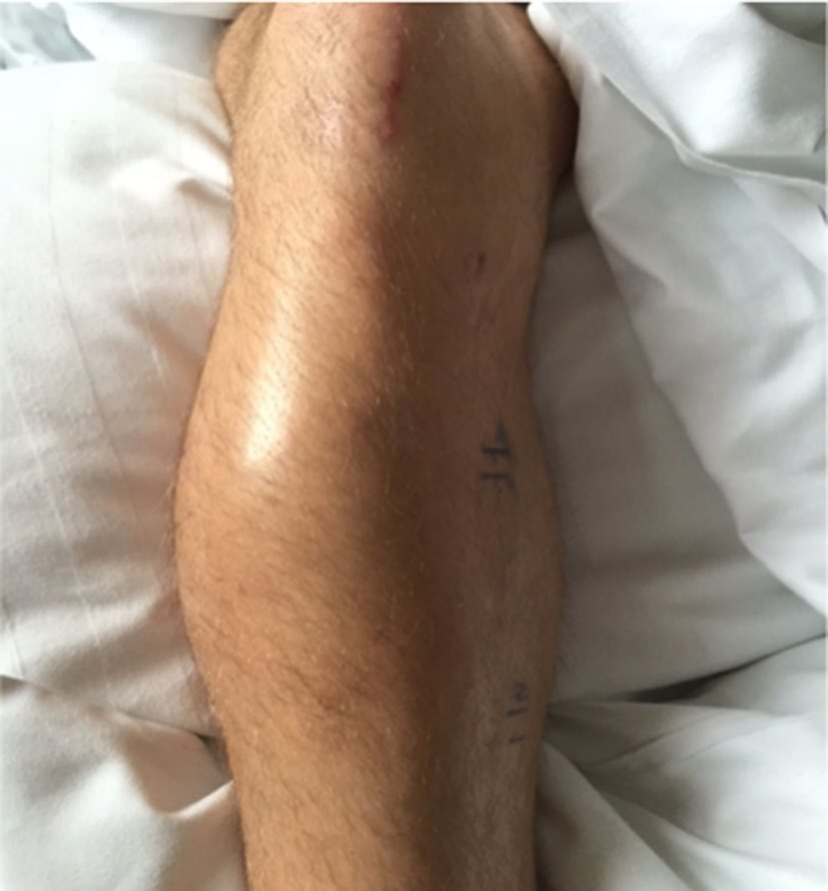
Recurrent swelling of the right lower leg 10 weeks after initial trauma

**Fig. 3:**
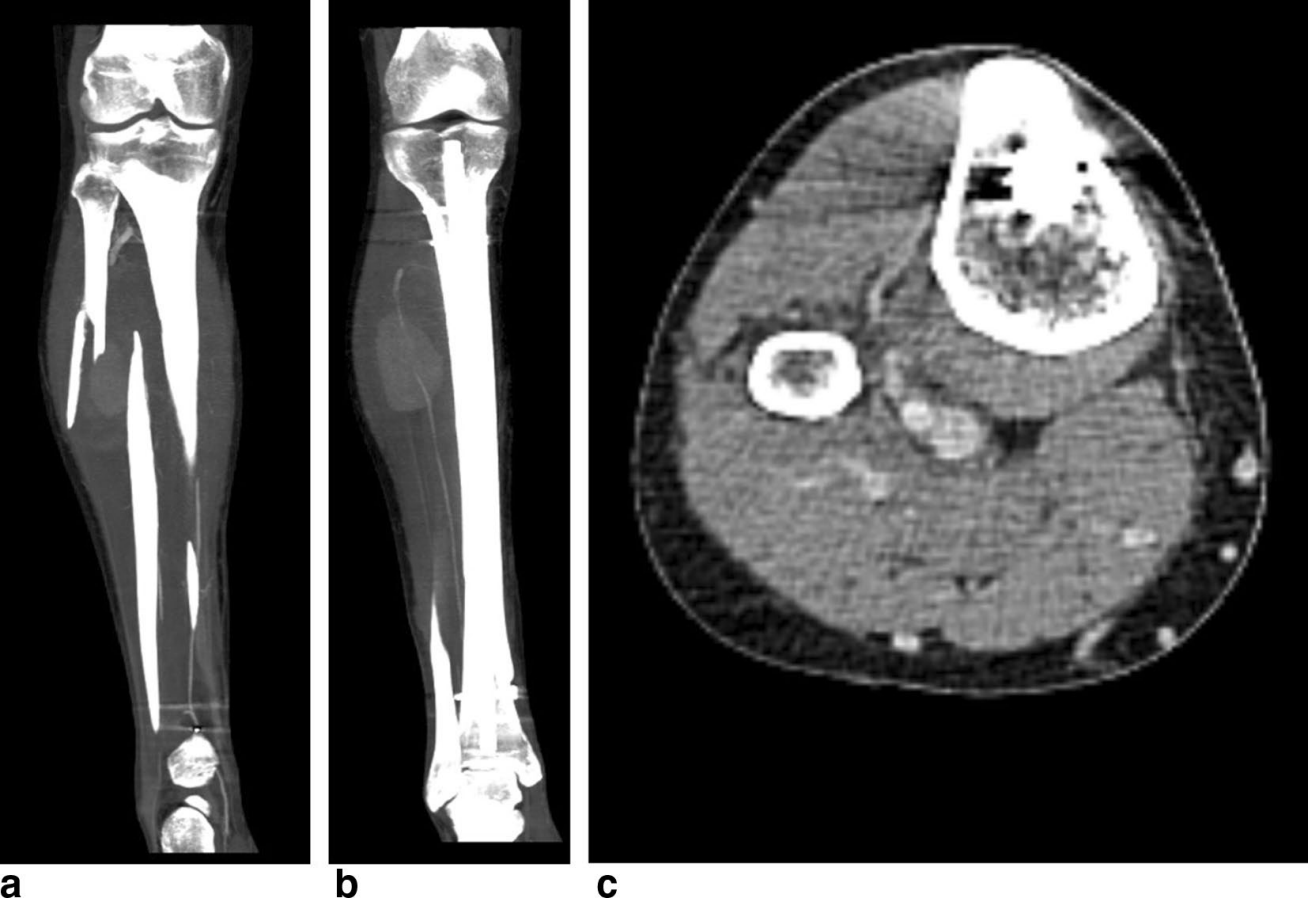
CT angiography of the lower leg showing the pseudoaneurysm (**a**) at the level of proximal screw insertion (**b**, **c**)

**Fig. 4:**
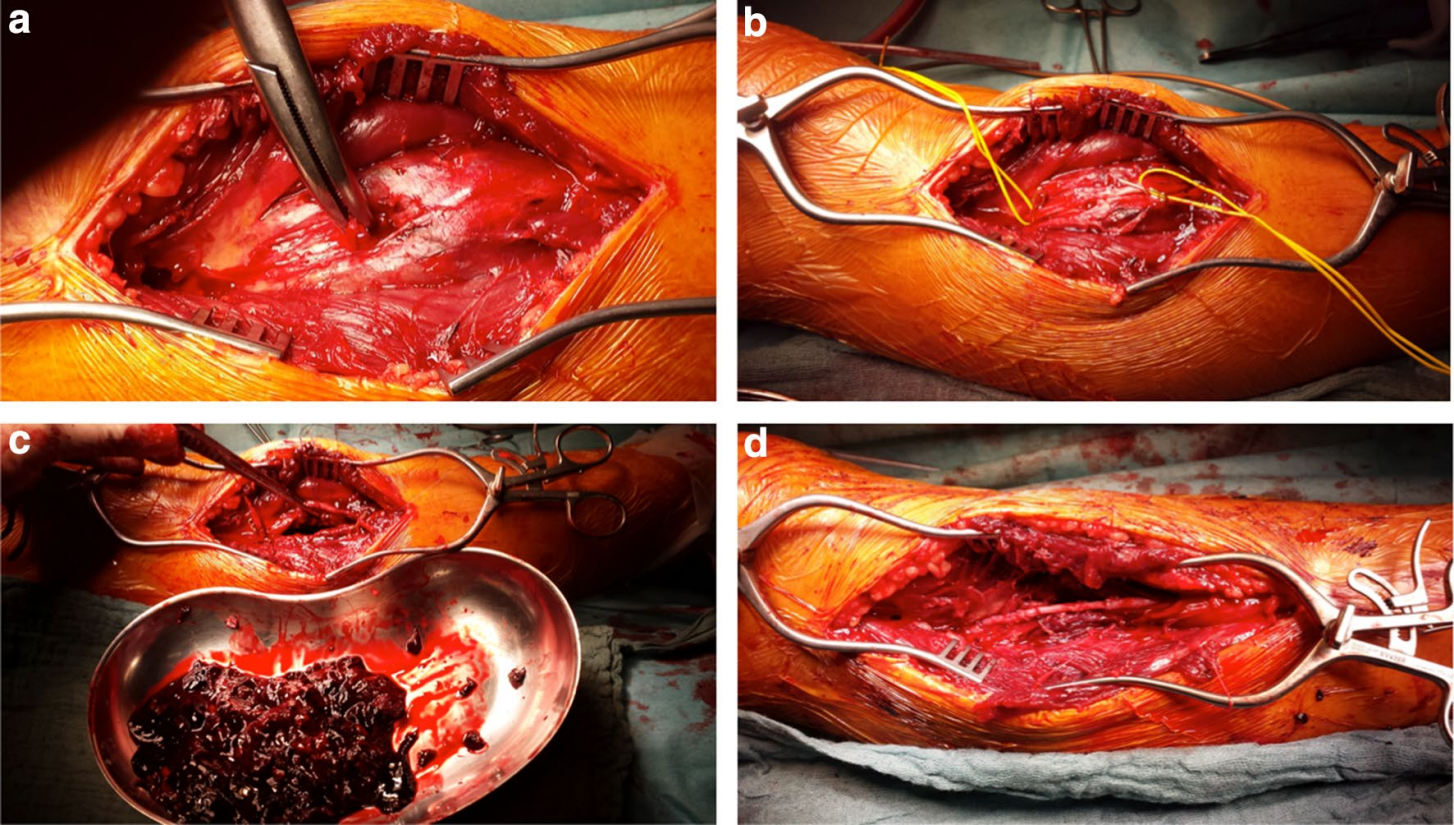
Intraoperative situs showing the membrane of the pseudoaneurysm (**a**). The peroneal nerve is marked (*yellow* vessel loop) (**b**). The pseudoaneurysm is resected (**c**). Bridging of the anterior tibial artery with an autograft of the greater saphenous vein (**d**)

**Fig. 5:**
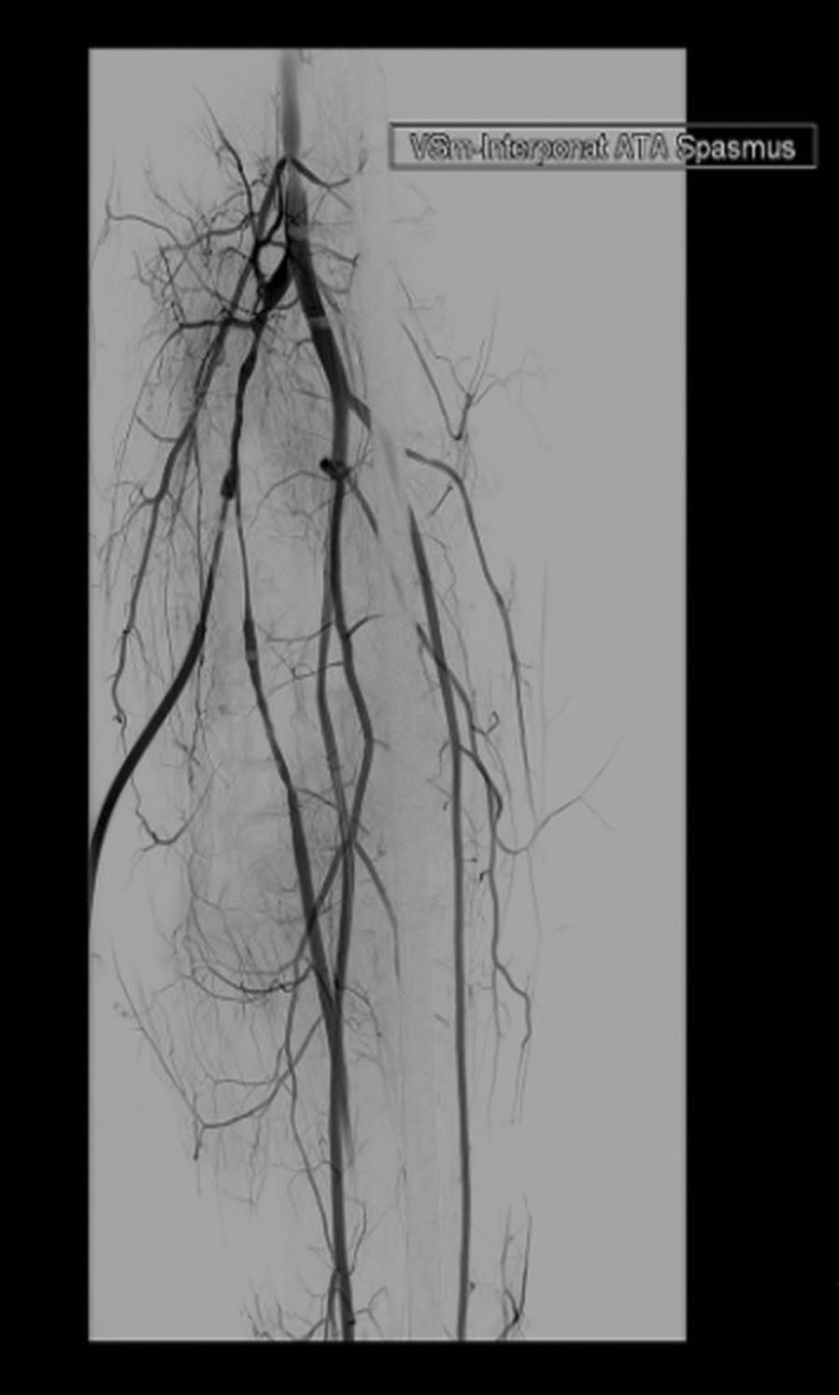
Intraoperative angiography showing normal blood flow over the repaired vessel defect

**Fig. 6:**
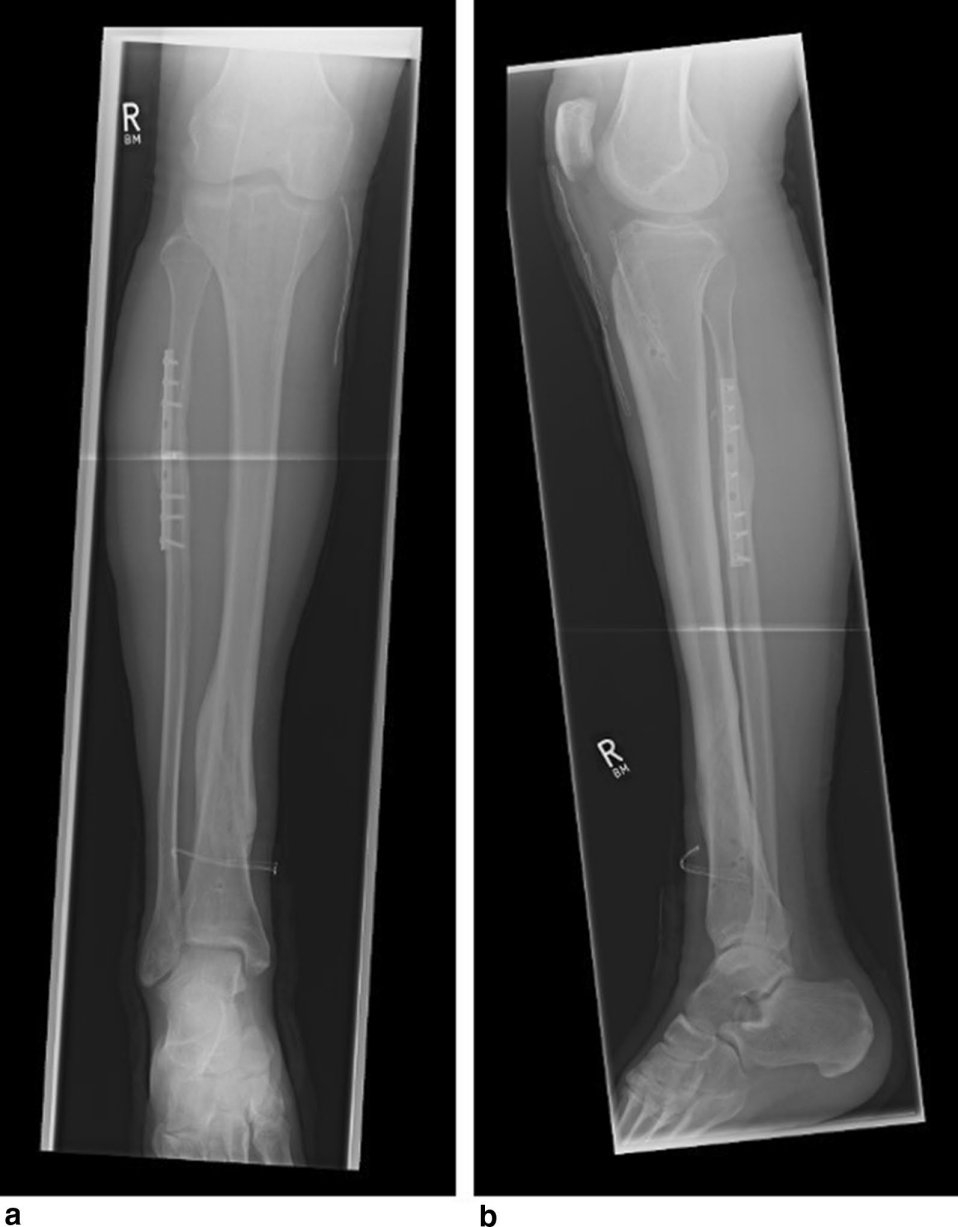
Anterio-posterior (**a**) and medio-lateral (**b**) radiographs 1 year after surgery, showing complete recovery of the tibial fracture and the remaining plate for fibula fracture reduction

Four weeks later, the patient was readmitted for reoccurring swelling of the proximal lower leg and additional pain and numbness of his dorso-medial foot (Fig. 2). Due to the intermittent nature of his complaints combined with neurologic symptoms, a CT angiography was performed which revealed a pseudoaneurysm of the anterior tibial artery in close proximity to the level of the proximal interlocking screw insertion (Fig. 3). Subsequently, surgery for vascular repair and fracture reduction of the fibula was performed. The vascular surgeons resected the aneurysm’s sac entirely and bridged the anterior tibial artery using the greater saphenous vein as an autologous graft (Fig. 4). Intraoperative angiography (Fig. 5) and postoperative Doppler ultrasound showed normal arterial blood flow. The fibular fracture was treated with standard AO/ASIF plating. Unfortunately, the patient’s peroneal nerve was affected intraoperatively. Electromyography confirmed a proximal peroneal paralysis with paresthesia of the dorsal foot and impaired dorsiflexion. After the patient was discharged, regular follow-up examinations showed improving peroneal nerve function and bone consolidation. One year after the initial fracture, the tibial nail was removed (Fig. 6), and the function of the peroneal nerve had recovered completely.

## Conclusion

While formation of pseudoaneurysms of the femoral artery after femoral nailing is a well-known surgical complication, pseudoaneurysms of the anterior tibial artery after tibial nailing are rare and thus often overlooked. Inamdar et al. [14] reported a single case of a 30-year-old patient who underwent surgical placement of an intramedullary interlocking nail after a proximal tibial fracture. Similar to our case, the patient developed a pseudoaneurysm of the anterior tibial artery at the level of the proximal medial–lateral screw insertion. In our patient, however, the symptoms did not occur immediately after surgery, but presented after weeks of an asymptomatic interval, impeding the diagnosis.

Usage of proximal interlocking screws, therefore, should be indicated restrictively. For prevention of complications, oblique locking screws should be preferred over medial–lateral locking screws. When inserting the screw, the surgeon should apply great caution while keeping in mind the anatomical course of the anterior tibial artery that originates from the popliteal artery, then passes from the backside through the proximal interosseous membrane, and descends in close proximity to the fibula into the anterior compartment of the leg.

One has to take into account that the dislocated fibula fracture as well could have had an impact on the development of the pseudoaneurysm. However, the few case reports regarding this specific complication describe the posterior tibial artery as the affected vessel rather than the anterior tibial artery [5, 15, 16].

During the postoperative course, swellings are often misinterpreted or underestimated as a physiological reaction. The assumption of pseudoaneurysm, for example, as a deep vein thrombosis, can lead to severe complications such as irreversible muscle atrophy of the lower leg as reported by Tylliankis et al. [4]. To avoid a delay in diagnosis and adequate therapy, postoperative recurrent swellings should be examined thoroughly with early performance of imaging diagnostics such as ultrasound, CT angiography, or MR angiogram in order to rule out rare, yet severe complications such as arterial pseudoaneurysms.

When performing vascular repair, the fibular fracture should be addressed simultaneously if severely dislocated. Peroneal nerve lesion is a complication that might occur as consequence of this surgical intervention or caused by the pseudoaneurysm itself. As seen in our patient, however, complete functional recovery of the nerve within 1 year is possible.

This case emphasizes that pseudoaneurysms pose a rare, yet serious complication after tibial nailing. Thorough examination and early diagnostic escalation using ultrasound, CT angiography, or MR angiogram are necessary to prevent a delay in treatment of this rare complication.

## Consent

Written informed consent was obtained from the patient for publication of this case report and any accompanying images.

## Abbreviations

AO: Arbeitsgemeinschaft für Ostheosynthese
ASIF: Association for the Study of Internal Fixation
CT: computed tomography
MR: magnetic resonance
